# Relationship between triglyceride glucose-body mass index and coronary artery calcium score in maintenance hemodialysis patients

**DOI:** 10.3389/fmed.2024.1478090

**Published:** 2024-12-13

**Authors:** Zexi Jiang, Jinhua Zhu, Hong Ding, Lihong Yan, Ruoxin Chen, Bin Wang, ZuoLin Li, Hong Liu

**Affiliations:** ^1^Institute of Nephrology, Zhongda Hospital, School of Medicine, Southeast University, Nanjing, China; ^2^Institute of Nephrology, People’s Hospital of Yangzhong City, Zhenjiang, Jiangsu, China

**Keywords:** triglyceride glucose-body mass index, coronary artery calcification, cardiovascular disease, maintenance hemodialysis, insulin resistance

## Abstract

**Background:**

This study investigated the association between coronary artery calcification (CAC) and triglyceride glucose-body mass index (TyG-BMI) in patients receiving maintenance hemodialysis (MHD).

**Methods:**

We used computed tomography (CT) to assess coronary artery calcification score (CACS) using the Agatston method. The TyG index was multiplied by BMI to derive the TyG-BMI index. Ordinal logistic regression models were used to analyze the relationship between TyG-BMI and CAC. The dose-response relationship was evaluated using restricted cubic spline regression. Weighted Quantile Sum regression was used to explore the weight of the TyG-BMI index components.

**Results:**

Based on the TyG-BMI, 219 patients with MHD were stratified into three groups. The TyG-BMI index was shown to be an independent risk factor for CACS by multivariate ordinal logistic regression analysis (odds ratio, 1.011; [95% confidence interval, 1.002–1.021]; *P* = 0.021). The relationship between TyG-BMI and lg (CACS + 10) was linear (*P*-overall = 0.023, *P*-non-linear = 0.412). Body mass index (BMI) had the highest weight (0.566) when weights were assigned to the three components of TyG-BMI. In the non-diabetes and diabetes subgroups, TyG-BMI and lg (CACS + 10) did not exhibit a significant non-linear relationship.

**Conclusion:**

TyG-BMI and CAC were independently positively correlated in patients undergoing MHD. These findings suggest that assessing TyG-BMI as a valuable tool for identifying the risk of CAC in patients with MHD.

## 1 Introduction

In China, cardiovascular disease (CVD) accounts for 20.2% of all hemodialysis patient deaths (1), making it the main cause of death in this demographic (2). Coronary artery calcification (CAC), a common complication and pathological change that occurs during hemodialysis (3), is an independent predictor of cardiovascular events (4). In Chinese maintenance hemodialysis (MHD), the prevalence of CAC is 71.8% (5) and it is closely associated with fatal and non-fatal CV events (5, 6). To attach essential significance to the diagnosis and treatment of CAC in patients on MHD, it is important to discover straightforward and reliable indicators for evaluating CAC.

Insulin resistance (IR) is one of the biggest risk factors for CVD (7), which also serves as the pathological foundation of CVD (8). It has been demonstrated that there is a clear correlation between IR and the occurrence of CAC, who have high IR levels having a 1.7-folds increased chance of developing CAC (9). IR increases lipids, blood sugar, and inflammatory indicators; which in turn prevent insulin’s anti-atherosclerotic effects and promote vascular calcification (10, 11). IR is widespread among MHD patients, not only in those with diabetes but also in those without diabetes (12). Compared to the homeostatic model assessment for insulin resistance (HOMA-IR), the triglyceride glucose-body mass index (TyG-BMI) combines blood glucose, lipids, and body mass index (BMI), making it easier to implement clinically. Furthermore it has a higher diagnostic value in identifying IR (13–15).

The TyG-BMI index and CVD (16, 17) as well as the severity of coronary artery disease (CAD) (18) have been found to be significantly correlated in previous studies. However, the association between TyG-BMI and CAC has not been well studied, with only one study suggesting a moderate prognostic effect of TyG-BMI on CAC progression in the general population (19). The link between TyG-BMI and CAC in patients on MHD is still unclear. Clarifying the association between TyG-BMI and CAC will help assess the risk of IR-related artery calcification in patients undergoing MHD and provide a new predictor of the risk of cardiovascular events in MHD patients.

Therefore, based on a single-center cross-sectional study of MHD patients in China, our research explored the relation between TyG-BMI index and CAC.

## 2 Materials and methods

### 2.1 Study design and participants

**FIGURE 1:**
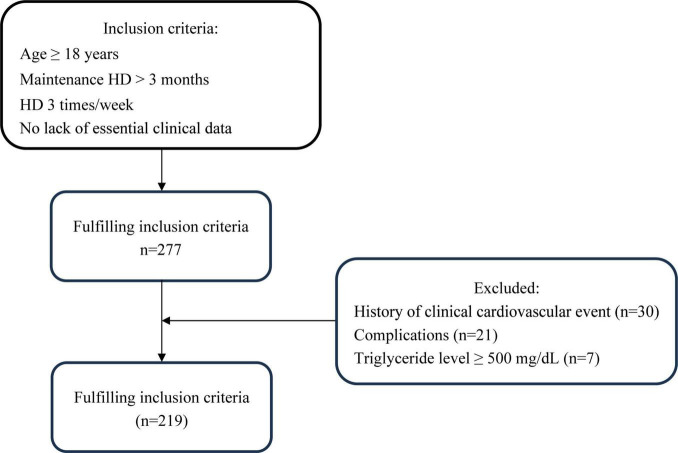
Enrollment flowchart for this study. HD, hemodialysis.

This cross-sectional prospective study included patients at Yangzhong People’s Hospital who were receiving stable hemodialysis. The inclusion criteria were the same as those in our previous study (20): (1) age > 18 years; (2) stable hemodialysis for > 3 months, 3 times weekly, 4 h each time; and (3) no lack of essential clinical data. Exclusion criteria were: (1) history of CAD, pacemaker, or defibrillator implantation; (2) complications with malignant tumor, severe heart failure (NYHA class IV), serious infection, connective tissue disease, blood system disease, severe cognitive impairment or mental illness; (3) pregnancy or lactation; (4) increased triglyceride level (≥ 500 mg/dL) (21). The 277 individuals in the Yangzhong People’s Hospital who underwent MHD from June 2022 to September 2022 were enrolled in this study. A flowchart of the patient selection process is shown in Figure 1. This study adhered to the principles of the Declaration of Helsinki, and it was approved by the Yangzhong People’s Hospital’s Ethics Committee (Approval No.: 2022053). Informed consent was obtained from all patients.

### 2.2 Data collection

Trained interviewers obtained data on demographic characteristics and health-related factors using a standardized procedure. The demographic characteristics included age and sex. Health-related factors included duration and modality of dialysis, self-reported medical history (diabetes and hypertension), use of antihypertensive or antidiabetic medications, current smoking status, and alcohol consumption. A history of hypertension or usage of antihypertensive drugs was defined as hypertension. Diabetes was defined as having a history of diabetes or use of antidiabetic medications. Regarding lifestyle measures, tobacco use was regarded as currently consuming at least one cigarette per day, and alcohol consumption was regarded as currently drinking more than three times per week. Clinical data were retrieved from electronic medical records, including levels of fasting blood glucose (FBG), triglycerides (TG), total cholesterol (TC), high-density lipoprotein cholesterol (HDL-C), low-density lipoprotein cholesterol (LDL-C), hemoglobin, albumin, calcium, phosphorus, magnesium, uric acid (UA), C-reactive protein (CRP), and parathyroid hormone (PTH). The triglyceride–glucose (TyG) index was calculated as ln (triglyceride [mg/dL] × glucose [mg/dL]/2) (22) and BMI was calculated as weight (kg) divided by height squared (m^2^). TyG-BMI was calculated as TyG multiplied by BMI (13).

### 2.3 Coronary artery calcium scoring (Agatston method)

Electron-beam computed tomography (CT) was performed using a 256-slice CT scanner (Revolution CT; GE Healthcare, Milwaukee, WI, USA) (20), Calcium scoring scans prospectively prompted by electrocardiography were conducted at 75% (heart rate < 65 beats per minute) or 45% (heart rate ≥ 65 beats per minute). Two experienced radiologists manually identified lesion locations on the CT scans using specially designed software (Smart Score 4.0; GE Healthcare) (20) after uploading the image data on a workstation (AW4.7; GE Healthcare). CAC was scored according to the Agatston method (23) as previously described. Patients were categorized into low CAC (0 ≤ CACS < 100), moderate CAC (100 ≤ CACS < 400), and high CAC (CACS ≥ 400) (24).

### 2.4 Statistical analysis

When comparing baseline data for normally distributed variables, one-way analysis of variance (ANOVA) was used to show the results as mean ± standard deviation. The median and interquartile range were used to express non-normally distributed data, while the Mann-Whitney U test was used for between-group comparisons. Counts and percentages were used to characterize categorical variables, and the chi-square test was used to compare them. Using coronary artery calcification score (CACS) as a continuous numerical variable, the association between CACS and clinical indicators was examined using Spearman’s correlation. Furthermore, Univariate and multivariate ordinal logistic regression analyses were performed to analyze the relationship between TyG-BMI and CACS. Additionally, a restricted cubic spline (RCS) regression models fitted for linear regression models was used to explore the linear relationship between TyG-BMI and lg (CACS + 10) with a four-knot model. TyG-BMI was derived from three variables (FBG level, TG level and BMI) with consistent directional effects. To determine the weights assigned to the three components and quantify each contribution to lg (CACS + 10), a weighted quantile sum (WQS) regression model (25, 26) was used to construct a weighted index, stratifying the combined effects into quartiles. The dataset was randomly divided into 40% for validation and 60% for training, and bootstrap resampling was performed with 1,000 iterations (27). Higher weights indicate greater significance of the respective indices in predicting lg (CACS + 10). Finally, subgroup analysis was performed using diabetes history as the basis. The R statistical software packages^1^ (The R Foundation) and SPSS software (version 26.0, SPSS, Chicago, IL, USA) were used for the statistical analyses, with *P* < 0.05 regarded as statistically significant.

## 3 Results

### 3.1 Baseline characteristics

A total of 219 patients undergoing MHD from Yangzhong People’s Hospital were enrolled in this study (89 women and 130 men). Table 1 presents the baseline characteristics of the patients. The average age was 57.88 ± 1.63 years, while the median duration of dialysis was 42.00 (19.00–78.00) months. Significant differences were noted among the different TyG-BMI groups in terms of sex, tobacco use, alcohol consumption, diabetes, FBG, TG, TC, HDL-C, LDL-C, albumin, phosphorus, UA, CRP, and CACS (*P* < 0.05). Notably, CAC and CACS increased with higher TyG-BMI.

**TABLE 1:** Characteristics of TyG-BMI index tertiles in the participants.

Variables	Total (*n* = 219)	TyG-BMI index			
		**Tertile 1 (*n* = 73)**	**Tertile 2 (*n* = 73)**	**Tertile 3 (*n* = 73)**	***P***
TyG-BMI	192.82 (167.87∼226.36)	162.11 (151.87∼168.05)	192.82 (185.10∼201.07)	239.16 (225.56∼254.36)	
Male sex, *n* (%)	130 (59.36%)	32 (43.84%)	47 (64.38%)	51 (69.86%)	0.003
Age (years)	57.88 ± 1.63	58.23 ± 3.19	58.00 ± 2.89	57.41 ± 2.48	0.917
BMI, (kg/m2)	22.65 ± 0.47	19.16 ± 0.39	22.64 ± 0.47	26.16 ± 0.56	< 0.001
Tobacco use, *n* (%)	61 (27.85%)	10 (13.70%)	25 (34.25%)	26 (35.62%)	0.004
Alcohol consumption, *n* (%)	42 (19.18%)	7 (9.59%)	15 (20.55%)	20 (27.40%)	0.022
Duration of dialysis (months)	42.00 (19.00–78.00)	42.00 (18.50–102.00)	44.00 (20.50–78.00)	39.00 (19.00–70.00)	0.721
SBP (mmHg)	145.96 ± 3.04	149.68 ± 5.56	144.75 ± 4.56	143.44 ± 5.73	0.219
DBP (mmHg)	78.56 ± 1.55	79.70 ± 2.83	78.41 ± 2.58	77.56 ± 2.70	0.535
Pulse pressure (mmHg)	64.00 (52.00–79.00)	67.00 (57.00–84.00)	67.00 (51.00–80.00)	62.00 (49.50–76.50)	0.221
**Comorbidities**					
Hypertension, *n* (%)	197 (89.95%)	65 (89.04%)	64 (87.67%)	68 (93.15%)	0.518
Diabetes, *n* (%)	68 (31.05%)	16 (21.92%)	22 (30.14%)	30 (41.10%)	0.005
**Laboratory parameters**					
FBG, (mmol/L)	4.90 (4.30–6.20)	4.70 (4.20–5.35)	4.80 (4.20–6.05)	5.40 (4.60–8.15)	0.002
TG, (mmol/L)	1.39 (0.88–2.31)	1.02 (0.64–1.42)	1.22 (0.86–2.13)	2.33 (1.58–3.10)	< 0.001
TyG index	8.60 (8.18∼9.21)	8.27 (7.75∼8.68)	8.47 (8.17∼9.09)	9.25 (8.80∼9.86)	< 0.001
TC, (mmol/L)	4.29 ± 0.14	4.26 ± 0.25	4.07 ± 3.86	4.53 ± 0.28	0.029
HDL-C, (mmol/L)	1.06 (0.90–1.31)	1.30 (1.12–1.43)	1.03 (0.87–1.23)	0.95 (0.85–1.05)	< 0.001
LDL-C, (mmol/L)	2.10 ± 0.10	1.99 ± 0.18	1.99 ± 0.15	2.32 ± 0.19	0.009
Hemoglobin, (g/L)	105.00 (92.00–116.00)	102.00 (89.50–113.50)	105.00 (92.00–118.50)	106.00 (95.50–117.50)	0.164
Albumin, (g/L)	41.90 (39.80–44.60)	40.90 (39.30–42.65)	42.40 (39.90–45.00)	42.60 (40.45–44.90)	0.017
Calcium, (mmol/L)	2.15 (2.07–2.28)	2.17 (2.08–2.29)	2.13 (2.04–2.26)	2.13 (2.07, 2.28)	0.547
Magnesium, (mmol/L)	1.18 (1.10–1.29)	1.19 (1.09–1.28)	1.20 (1.14–1.29)	1.16 (1.08–1.31)	0.344
Phosphorus, (mmol/L)	1.86 ± 0.07	1.72 ± 0.11	1.82 ± 0.11	2.03 ± 0.15	0.002
UA, (μmol/L)	411.64 ± 12.7	383.03 ± 18.43	406.63 ± 22.78	445.26 ± 23.03	< 0.001
CRP, (mg/L)	0.66 (0.50–5.28)	0.50 (0.50–2.12)	0.50 (0.50–3.94)	2.72 (0.54–8.56)	< 0.001
PTH, (pg/mL)	184.10 (93.80–349.90)	151.00 (90.05–293.85)	181.20 (88.45–352.30)	218.80 (115.45–402.50)	0.117
CACS	394.78 (44.45–1,041.10)	269.61 (38.64–675.32)	394.18 (29.11–1,117.61)	515.18 (178.81–1,633.37)	0.023
0 ≤ CACS < 100	66 (30.14%)	25 (34.25%)	28 (38.35%)	13 (17.81%)	0.017
100 ≤ CACS < 400	46 (21.00%)	18 (24.65%)	9 (12.33%)	19 (26.03%)	0.082
400 ≤ CACS	107 (48.86%)	30 (41.10%)	36 (49.32%)	41 (56.16%)	0.190

Values are *n* (%), mean ± standard deviation or median (interquartile range). SBP, systolic blood pressure; DBP, diastolic blood pressure; FBG, fasting blood glucose; TG, triglyceride; TC, total cholesterol; HDL-C, high-density lipoprotein cholesterol; LDL-C, low-density lipoprotein cholesterol; UA, uric acid; CRP, C-reactive protein; PTH, parathyroid hormone; CACS, coronary artery calcification score.

### 3.2 Correlations between TyG-BMI index and CACS

According to Spearman’s correlation analysis, TyG-BMI (*P* = 0.007), age, tobacco use, duration of dialysis, and FBG, HDL-C, hemoglobin, albumin, and CRP levels were found to be associated with CACS (Table 2).

**TABLE 2:** Correlations between the CACS and risk factors.

Variables	*r*	*P*
Male sex, *n* (%)	0.119	0.079
Age (years)	0.210	0.002
Tobacco use, *n* (%)	0.173	0.010
Alcohol consumption, *n* (%)	0.058	0.391
Duration of dialysis (months)	0.330	< 0.001
SBP (mmHg)	0.020	0.766
DBP (mmHg)	−0.125	0.064
Pulse pressure (mmHg)	0.114	0.093
Hypertension, *n* (%)	0.055	0.419
Diabetes, *n* (%)	0.070	0.305
FBG, (mmol/L)	0.139	0.039
TG, (mmol/L)	0.107	0.115
TC, (mmol/L)	−0.034	0.619
HDL-C, (mmol/L)	−0.255	< 0.001
LDL-C, (mmol/L)	0.015	0.820
Hemoglobin, (g/L)	−0.141	0.037
Albumin, (g/L)	−0.241	< 0.001
Calcium, (mmol/L)	−0.001	0.989
Magnesium, (mmol/L)	−0.078	0.252
Phosphorus, (mmol/L)	0.030	0.657
UA, (μmol/L)	0.040	0.561
CRP, (mg/L)	0.173	0.010
PTH, (pg/mL)	0.090	0.184
TyG-BMI	0.182	0.007

SBP, systolic blood pressure; DBP, diastolic blood pressure; FBG, fasting blood glucose; TG, triglyceride; TC, total cholesterol; HDL-C, high-density lipoprotein cholesterol; LDL-C, low-density lipoprotein cholesterol; UA, uric acid; CRP, C-reactive protein; PTH, parathyroid hormone; TyG-BMI, glucose-body mass.

Table 3 indicates that a strong correlation (odds ratio, 1.007; [95% confidence interval, 1.001–1.014]; *P* = 0.030) was found between the TyG-BMI score and CACS by univariate ordinal regression analysis. Even after adjusting for possible confounding covariates, including age, sex, tobacco use, alcohol consumption, diabetes, hypertension, duration of dialysis, diastolic blood pressure, pulse pressure, FBG, HDL-C, hemoglobin, albumin and CRP, multivariate ordinal logistic regression analysis revealed that the TyG-BMI index remained significantly associated with CACS (odds ratio, 1.011; [95% confidence interval, 1.002–1.021]; *P* = 0.021).

**TABLE 3:** Multivariate ordinary logistic regression analysis of the relationship of TyG-BMI index to CAC.

TyG-BMI index	β	SE	OR	95% CI	Wald	*P*
Unadjusted	0.007	0.003	1.007	1.001, 1.014	4.693	0.030
Model 1	0.007	0.004	1.007	1.000, 1.014	4.282	0.039
Model 2	0.007	0.004	1.007	1.000, 1.014	3.987	0.046
Model 3	0.011	0.005	1.011	1.002, 1.021	5.773	0.021

Model 1 adjusted for age, sex; Model 2 adjusted for age, sex, tobacco use, alcohol consumption, hypertension, diabetes; Model 3 adjusted for age; sex; tobacco use; alcohol consumption; diabetes; hypertension; duration of dialysis; diastolic blood pressure; pulse pressure; fasting blood glucose; high-density lipoprotein cholesterol; hemoglobin; albumin and C-reactive protein. SE, standard error; OR, odds ratio; CI, confidence interval.

### 3.3 Linear relationship between TyG-BMI index and CACS

**FIGURE 2:**
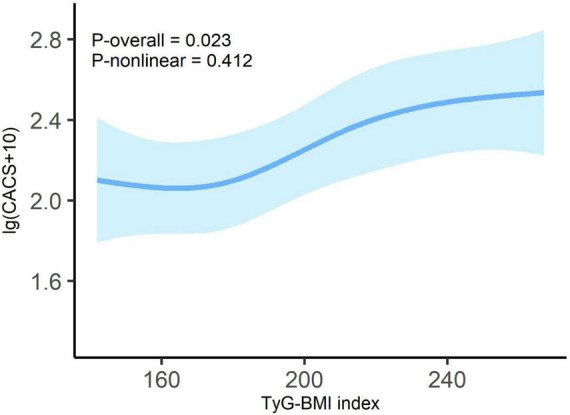
Dose-response relationship between TyG-BMI index and lg (CACS + 10) in overall patients. Adjusted for age; sex; tobacco use; alcohol consumption; diabetes; hypertension; duration of dialysis; diastolic blood pressure; pulse pressure; fasting blood glucose; high-density lipoprotein cholesterol; hemoglobin; albumin and C-reactive protein. Odds ratios are indicated by solid lines and 95% CIs by shaded areas.

As presented in Figure 2, after controlling for covariates, we observed a linear dose-response relationship between TyG-BMI and lg (CACS + 10) (*P*-overall = 0.023, *P*-non-linear = 0.412).

### 3.4 WQS analysis

**FIGURE 3:**
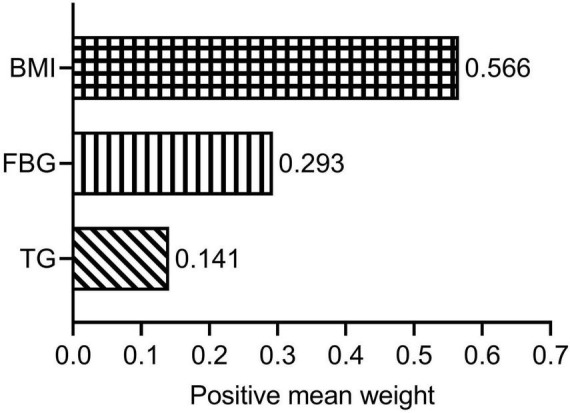
Estimated weights assigned to TyG-BMI with the WQS model. WQS models were adjusted for age; sex; tobacco use; alcohol consumption; diabetes; hypertension; duration of dialysis; diastolic blood pressure; pulse pressure; high-density lipoprotein cholesterol; hemoglobin; albumin and C-reactive protein. BMI, body mass index; FBG, fasting blood glucose; TG, triglyceride; WQS, weighted quantile sum.

Using the WQS regression model, we explored the impact of the three components (FBG, TG, and BMI) of the TyG-BMI index on lg (CACS + 10) after adjusting for possible covariates. The WQS index was significantly associated with lg (CACS + 10) (β = 0.205; [95% confidence interval, 0.039–0.371]; *P* = 0.018). Figure 3 illustrates the weights of the contributing components of TyG-BMI, with BMI being the primary contributor (weight = 0.566).

### 3.5 Subgroup analysis

**FIGURE 4:**
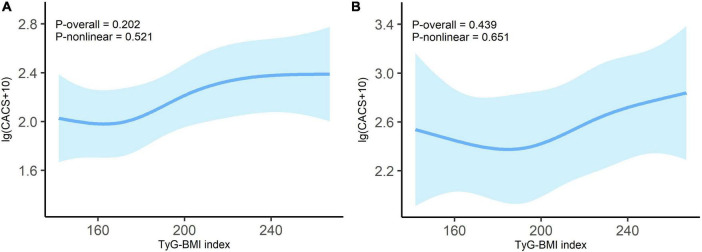
Dose-response relationship between TyG-BMI index and lg (CACS + 10) in non-diabetes **(A)** and diabetes **(B)** patients. Adjusted for age; sex; tobacco use; alcohol consumption; hypertension; duration of dialysis; diastolic blood pressure; pulse pressure; fasting blood glucose; high-density lipoprotein cholesterol; hemoglobin; albumin and C-reactive protein. Odds ratios are indicated by solid lines and 95% CIs by shaded areas.

To investigate whether there was a stable linear relationship between TyG-BMI and CACS in the non-diabetes and diabetes subgroups, we employed the RCS model. The interaction analysis (*P* = 0.638) suggested that the relationship between TyG-BMI index and CACS was independent of diabetes status. As shown in Figure 4, there was no significant non-linear relationship between the TyG-BMI index and lg (CACS + 10) in both non-diabetes (*P*-overall = 0.202, *P*-non-linear = 0.521) and diabetes patients (*P*-overall = 0.439, *P*-non-linear = 0.651) with all potential covariates fully adjusted.

## 4 Discussion

This study proposes that in patients on MHD, TyG-BMI is significantly positively correlated with CAC, even after adjusting for possible clinical risk factors. A linear dose-response association was observed between TyG-BMI and lg (CACS + 10). When weights were assigned to the three TyG-BMI components, BMI emerged as the primary contributor. In the diabetes and non-diabetes subgroups, there was no significant non-linear relationship between TyG-BMI and lg (CACS + 10).

In the general population, CAC primarily involves calcification of atherosclerotic plaques (28). Large-scale longitudinal observational studies conducted in the United States and Europe have demonstrated a significant relationship between CAC and major cardiovascular outcomes in the general individuals (29, 30). In dialysis patients, calcified deposits within the intima lead to atherosclerosis, whereas deposits within the media contribute to arteriosclerosis (28). CAC is highly associated with MHD mortality, independent of traditional and non-traditional risk factors (31). Consequently, studying the risk factors related to CAC in MHD patients is of considerable significance.

IR refers to the impaired or decreased capacity of insulin-dependent organs and tissues to respond to endogenous and exogenous insulin and is characterized by impaired glucose uptake, defects in oxidation, and the inability to restrain lipid oxidation (7). It is widely acknowledged that decreased peripheral tissue glucose consumption in IR is intimately linked to the deterioration of renal function (32). Prior researches have tried to clarify the underlying mechanisms of IR in individuals with CKD. First, bacterial DNA can move from the gut into the bloodstream due to a weakened intestinal epithelial barrier in individuals with CKD (33). The cytokines generated during this chronic inflammation can also directly trigger macrophage migration and disrupt insulin signaling pathways (34). Second, IR can be brought on by metabolic dysfunction caused by elevated adipokines (such as leptin and adiponectin) in CKD patients (35). Third, IR may also be present in CKD patients who are deficient in vitamin D. Due to the fact that vitamin D insufficiency affects pancreatic β cells’ glucose-mediated insulin secretion (36). Furthermore, oxidative stress, anemia, metabolic acidosis, and other factors are all contributing factors to IR in CKD (35).

As the pathological basis of CVD (8), IR may lead to vascular calcification through multiple mechanisms, including elevated oxidative stress, endothelial dysfunction, and increased inflammatory cytokines (10, 11). In MHD patients, the chronic inflammatory state is mutually reinforcing and influences IR. Additionally, macrophages release inflammatory cytokines such TNF-α and IL-1, which have been shown to promote bone formation in vascular smooth muscle cells (9). Second, in IR, increased endothelial mediator production due to elevated serum insulin levels results in endothelial cell dysfunction, vascular smooth muscle cell migration and proliferation, and vascular calcification (11, 37). CAC in MHD patients is caused by a confluence including IR, chronic inflammation, oxidative stress, and dialysis toxins (38).

Compared with HOMA-IR, the new TyG-BMI index is a more favorable IR substitute (13, 39). In terms of detecting non-alcoholic fatty liver, the TyG-BMI had the best diagnostic ability when compared to other IR-related indicators including TyG index, TG/HDL-c and Visceral Adiposity index (39). According to a study with 11,149 general participants, TyG-BMI outperformed TyG, TyG-WC, and TyG-WHtR in terms of discriminatory power in predicting IR in both men and women (13). Besides, TyG-BMI has a strong diagnostic value for CAD (18) in addition to its beneficial value in IR-related diseases (13, 39). Stated differently, TyG-BMI is a convenient and dependable CVD indication as well (16). In the US population, the TyG-BMI has a strong correlation with CVD mortality and diagnostic value for overall CVD, HF, myocardial infarction, angina pectoris, and CHD (40). However, the relationship between TyG-BMI and CAC remains unknown. Only one study has reported a positive correlation between TyG-BMI and CAC progression in the general population (19). This is the first study to find a significant positive relationship between TyG-BMI and CAC in patients undergoing MHD. Multivariate regression analysis suggested that TyG-BMI was independently associated with CACS in MHD patients. The probability of CACS changing at least one level for every 10 units increase in TyG-BMI was 11%. We employed the RCS regression model to identify a linear association between TyG-BMI and lg (CACS + 10) to further decipher its relationship. One study showed that TyG-BMI linearly correlated with myocardial infarction, CHD, and total CVD (40). Our findings were also supported by a study from the China Health and Retirement Longitudinal Study, which found a linear relationship between cumulative average TyG-BMI and myocardial infarction, CHD, and total CVD (17). In addition, subgroup analysis of the non-diabetes and diabetes subgroups suggested that there was no significant non-linear association between TyG-BMI index and lg (CACS + 10). We further elucidated the relationship between TyG-BMI and lg (CACS + 10) using the WQS regression model, with BMI as the main contributor (0.566). Obesity is also highly linked to the risk of CVD in a variety of populations, according to numerous prior research (41–43). However, using WQS analysis, Huo et al. (26) found that TG had the highest weight when studying the relationship between TyG-BMI and stroke risk.

This study has a few limitations. First, our data were obtained from one single center with a relatively small sample size. Although the subgroups showed comparable tendencies to the overall, larger multicenter studies are warranted. Second, this was a clinical observational study; therefore, we could not determine the causal relationship between IR and CAC. Third, this study used only one baseline blood sample to collect TyG-BMI information; therefore, we could not assess the impact on CAC over time.

## 5 Conclusion

In this single-center, cross-sectional study, we found a linear relationship between TyG-BMI and CACS in patients with MHD. TyG-BMI assessment is a useful tool for determining the IR-related risk of CAC in patients with MHD. Nevertheless, larger multicenter cohort study with more sample size is required to further confirm these results.

## Data Availability

The datasets used and/or analyzed during the current study are available from the corresponding author on a reasonable request.

## Footnotes

1
http://www.r-project.org
